# Information Acquisition Ability and Consumer Preferences for Food-Label Attributes: Evidence from China’s Pork Market

**DOI:** 10.3390/foods15213996

**Published:** 2026-11-07

**Authors:** Lulu Tou, Jianhua Wang, May Chu

**Affiliations:** 1 China Academy for Rural Development, School of Public Affairs, Zhejiang University, Hangzhou 310058, China; lulutou@zju.edu.cn; 2 School of Business, Jiangnan University, Wuxi 214122, China; 3 Department of Public Administration and Policy, School of Government, University of Birmingham, Birmingham B15 2TT, UK

**Keywords:** food safety and sustainability, information acquisition ability, food labels, consumer preferences, item response theory, Sustainable Development Goals, SDG 12

## Abstract

The growing number and complexity of food-label schemes may undermine their effectiveness in promoting sustainable food consumption and incentivizing higher production standards, because consumers differ in their ability to acquire label information. This study examines how consumers’ information acquisition ability (IAA) shapes their preferences for pork-label attributes that signal different levels of food safety and production standards. Using survey and discrete choice experiment data from 844 consumers in East China, we first estimate latent IAA from eight information channels with an item response theory model and then incorporate it into a random parameters logit model. The results reveal an information bottleneck. Online social media is the easiest channel through which to obtain label information, but it differentiates sharply between consumers with different levels of acquisition ability, whereas institutional channels such as government departments and community noticeboards are the most difficult to use. Just over half of the respondents fall below the average level of acquisition ability, and only a small group can obtain label information from a wide range of channels. Consumers with higher IAA place greater value on Green, Organic and drug-free labels and somewhat less value on the entry-level Hazard-free certification, suggesting that basic safety signals become less distinctive as acquisition ability increases. These associations are most evident in households with minors. Improving consumers’ capacity to acquire credible label information may help translate demand for safer and more sustainable food into stronger market incentives for higher-standard production.

## 1. Introduction

Food quality and safety are central to sustainable food systems and to the United Nations Sustainable Development Goals (SDGs), particularly SDG 3 (Good Health and Well-Being) and SDG 12 (Responsible Consumption and Production). Yet foodborne illnesses remain a persistent global threat, causing an estimated 420,000 deaths annually [1,2] and imposing substantial economic costs through medical expenditures and productivity losses [3]. While SDG 3 underscores the need to reduce food-related health risks, SDG Target 12.8 emphasizes the information and awareness needed for sustainable lifestyles. Food labels can help connect these goals to consumer choice by conveying information about product safety and production standards. Their potential, however, depends on whether consumers can acquire and interpret the information they provide.

In response to food-safety concerns and growing attention to environmental sustainability, China has gradually developed an agricultural-product quality and safety certification system centered on Hazard-free (wugonghai), Green Food, and Organic certifications since the 1990s [4,5]. As early as the end of 2016, more than 100,000 products were covered by the country’s major agricultural certification and labeling programs [6]. Beyond certification marks, consumers encounter origin labels, traceability information, and other product claims in physical and online markets. The wider availability of these signals gives consumers more information about food safety and production standards, but their variety may also increase the effort required to find, distinguish, and interpret credible information [7,8]. In an era of label proliferation, a consumer’s true market power is not defined by whether information is present, but by their ability to acquire and identify such information, namely their information acquisition ability (IAA). This ability may shape how consumers evaluate pork products carrying different safety and production-standard signals.

This study investigates how IAA acts as an individual determinant of consumer preferences in China’s pork market. To examine this mechanism, we utilize a unique data window in 2017. The year 2017 marked a critical juncture in China’s digital transformation, as the digital economy was formally incorporated into the national policy agenda for the first time and the divergence between traditional media channels and emerging digital platforms continued to accelerate. This period therefore provides an ideal structural baseline, capturing an important transition in consumers’ information behavior from passive information reception to active acquisition. By identifying the mechanisms of IAA during this transformative period, we show how this latent ability relates to market demand and provide traceable evidence for understanding the structural heterogeneity of consumer choice behavior across different communication networks.

The challenge in existing research lies in the precise measurement of this acquisition ability. Earlier studies often relied on simple channel counts, which fail to distinguish between the qualitative differences in information sources [9,10]. This approach ignores the varying cognitive and technical thresholds—or entry barriers—of different media. To overcome this, we employ a two-parameter logistic (2PL) item response theory (IRT) model to measure IAA as a latent trait. The 2PL-IRT model is well suited to this purpose for three reasons. First, it estimates a difficulty and a discrimination parameter for each channel, so that channels are weighted according to how demanding they are and how well they distinguish between consumers, rather than being counted as equivalent [11]. Second, it places channels and consumers on a common scale and yields an ability score for each consumer, which makes it possible to compare consumers’ knowledge of certification labels across different levels of acquisition ability and to link this ability to their label preferences [12]. Third, it is designed for binary indicators, such as whether a consumer can obtain label information from a given channel, which matches the structure of our survey data. These properties allow us to treat IAA not as a fleeting market trend but as a stable behavioral characteristic that can be linked to consumers’ label preferences.

Furthermore, we integrate this latent trait into a random parameters logit (RPL) model within a discrete choice experiment (DCE) framework. Pork provides a particularly salient empirical setting for two reasons. First, pork production constitutes an important source of agricultural non-point source pollution in China, with its environmental burden arising notably from nutrient inputs associated with feed production and nutrient losses associated with manure management [13,14]. Improving the sector’s environmental performance is therefore consequential for China’s broader transition toward agricultural sustainability. Second, China’s pork market is shifting from traditional wet markets dominated by unchilled “hot meat” toward more standardized retail environments. In these modernized channels, the direct sensory evaluation of unbranded products increasingly gives way to formal credence signals. Consequently, food labels become the primary institutional mechanisms for mitigating information asymmetry, while consumers’ ability to acquire and identify these signals constitutes a key cognitive filter through which external information is transformed into individual utility. This raises a central yet underexplored question in the context of pork consumption: Is IAA associated with systematic differences in consumers’ valuation of different labels? Guided by this logic, we specifically test for a utility-driven crowding-out effect: as IAA increases, basic safety assurances may shift from significant utility-enhancing attributes to baseline expectations, causing their utility space to be replaced by the more rigorous safety and sustainability assurances promised by premium certifications.

The objective of this study is therefore to examine whether, and for which label attributes, consumers’ IAA is associated with their preferences for labeled pork products. Specifically, we address three research questions:

1. How do information channels—particularly digital versus traditional media—differ in their difficulty and discrimination as indicators of consumers’ information acquisition ability (IAA)?
2. Is higher IAA associated with greater utility from Green and Organic certifications and lower utility from the basic Hazard-free certification, consistent with a crowding-out of baseline safety signals?
3. Does the association between IAA and label preferences differ between consumers with and without prior experience of pork-safety incidents, and between households with and without minors?

By answering these questions, this study makes three contributions. First, it shifts the analysis of food labels from the provision of information to consumers’ ability to acquire it. Whereas most labeling studies examine how the presence, design or framing of a label affects preferences, we treat IAA as a latent consumer characteristic and show that it is systematically related to how consumers value labels of different stringency, which links research on bounded rationality with information economics in the food-safety context. Second, it makes a measurement contribution: rather than counting the channels a consumer uses, we estimate how difficult and how discriminating each channel is as an indicator of acquisition ability, which shows that easily accessible digital channels and hard-to-reach institutional channels play very different roles. Third, it provides evidence from a three-tier certification system that is relevant to other emerging economies, identifying the consumer segments—such as households with minors—in which acquisition ability is most strongly associated with demand for higher-standard labels. This evidence can inform targeted communication strategies and the design of label portfolios, helping to transmit demand for safer and more sustainable food to the production side.

## 2. Literature Review

Within the broader transition toward sustainable food systems, food safety has evolved from a public health mandate to a market-driven quality attribute, prompting a re-evaluation of how food quality and safety information reaches and is recognized by the end-user. While traditional economic models assume that more information is better, the modern digital landscape suggests that the value of a safety signal is contingent upon consumers’ ability to navigate heterogeneous information channels. This review synthesizes the shift from macro-level market failures to micro-level cognitive filters, providing the theoretical justification for a psychometric approach to consumer choice.

### 2.1. Information Asymmetry and the Signaling Role of Food Labels

The foundational challenge in food markets is information asymmetry [15]. Food safety is a credence attribute that consumers cannot verify even after consumption [16], and the sustainability of the underlying production process is even further removed from direct verification. Third-party certifications address this gap by acting as signals of attributes that consumers cannot observe themselves [17]. In China, such signaling is organized around a national certification hierarchy of Hazard-free, Green Food and Organic tiers [18], complemented by cues such as product traceability, origin information and drug-free claims.

A signal, however, conveys value only if consumers understand and trust it. In a cross-country European study, Grunert et al. [19] found that consumers’ understanding of sustainability labels was limited and that label use depended on both motivation and understanding. Janssen and Hamm [20] showed that willingness to pay for organic food differs substantially across certification logos with similar standards, highlighting the role of familiarity and trust rather than stringency alone. In China, consumers place the highest value on government-backed certification of food-safety attributes [21] and on government certification within pork traceability information [22], yet their trust varies considerably across information sources [23], and concerns about counterfeit labels and food fraud are widespread [24]. The value of a label is therefore not intrinsic to the certification itself but depends on what consumers know about it and where that knowledge comes from. This dependence becomes more consequential as label schemes proliferate. When multiple labels coexist and compete for attention, signal effectiveness depends not only on institutional availability but also on whether consumers can access and distinguish these signals [25,26], and the same certification may be more or less salient to different consumers depending on the channels through which they encounter label information. The analytical focus thus needs to move from the provision of label signals to consumers’ heterogeneous ability to acquire them.

### 2.2. Beyond Information Provision: The Cognitive Shift to IAA

From the perspective of bounded rationality, consumers are unlikely to access or attend to all available label information equally, because search costs, channel accessibility and channel quality constrain their engagement. This perspective motivates the concept of IAA, defined as consumers’ latent ability to access and identify relevant food-label information from heterogeneous channels [27]. IAA is related to, but distinct from, consumer knowledge. Research on information search shows that prior knowledge makes the acquisition of new information more efficient and directs attention to more diagnostic attributes [28], and in the organic food context, both objective and subjective knowledge are positively associated with more frequent use of information sources [29]. Whereas knowledge refers to what consumers already know, IAA captures their capacity to obtain such knowledge, which is likely to matter most where labels are numerous and unfamiliar. Research on the digital divide points in the same direction: as internet access becomes nearly universal, inequalities shift from physical access to differences in skills and usage, the so-called second-level digital divide [30,31]. China in 2017 fits this description, as the rapid expansion of digital platforms alongside traditional media widened differences in how consumers obtained food-related information. Moreover, because information overload can impair decisions when the volume of information exceeds consumers’ processing capacity [26], greater availability does not necessarily translate into better-informed choices. Together, these strands imply that the same information environment can yield very different effective information sets for different consumers, depending on whether label signals become visible and distinguishable to them [32]. Existing food-label studies, however, have rarely measured this ability directly; they typically rely on self-reported knowledge or treat information exposure as an experimental treatment [32,33].

### 2.3. Methodological Evolution: From Simple Counts to Latent Traits

Measuring IAA is not straightforward. Earlier studies typically used composite indices that count the number of channels a consumer uses [10]. Such indices assume that all channels are equally informative about the underlying ability, so that obtaining label information from social media counts the same as obtaining it from a government department, even though the two may differ considerably in how demanding they are to use. IRT offers an alternative. Originating in psychometrics, it models the probability that a respondent gives a positive response to an item as a function of the respondent’s latent trait and the item’s characteristics [11,12]. In the two-parameter model, each item has a difficulty parameter, which indicates the level of the trait at which a positive response becomes likely, and a discrimination parameter, which indicates how sharply the item separates respondents with lower and higher levels of the trait. In economics, IRT has been used to measure constructs that can be observed only through binary indicators, such as household wealth measured from asset ownership [10], where it captures differences between indicators that summated indices treat as equivalent. Abdul-Salam and Phimister [34] applied IRT to information channels, estimating the difficulty and discrimination of each channel. Applied to label information, the approach weights channels according to how informative they are about acquisition ability rather than treating them as interchangeable.

Like other measurement models, IRT rests on assumptions about the structure of the latent trait, and individual scores are estimated with some error [11]. Integrated choice and latent variable models address the latter issue by estimating the latent variable jointly with the choice model, but they are considerably more demanding to specify, identify and estimate [35]. A two-step approach, in which IAA is first estimated with IRT and then entered into the choice model, may be less statistically efficient, but it keeps the measurement of acquisition ability separate from, and interpretable independently of, the analysis of label preferences. This separation is particularly useful for the present study, whose first research question concerns the properties of the information channels themselves. For research that seeks both to compare information channels and to relate acquisition ability to label preferences, a two-step IRT approach therefore offers a suitable balance between statistical efficiency and transparency.

### 2.4. Preferences for Food Labels: The Hazard-Free-to-Organic Transition

China’s food-labeling system comprises multiple signals, with the multi-tier certification system constituting the core institutional framework for communicating differentiated standards [36,37]. Hazard-free certification serves as the baseline threshold for food safety, certifying that products comply with mandatory national requirements on pesticide and veterinary drug residues and on production-area conditions while permitting the regulated use of agrochemicals [21,38]. Green Food certification further restricts the types and quantities of agrochemical and veterinary inputs and requires environmental monitoring of the production area, thereby extending assurance beyond basic food safety toward broader sustainability considerations [39]. Organic certification, governed by the national standard GB/T 19630, is the most stringent: it excludes synthetic fertilizers, pesticides, growth promoters and genetically modified inputs, requires a conversion period, and more closely integrates food-safety assurance with environmentally oriented production principles [40]. The three tiers thus differ in their safety assurance, production standards and environmental requirements.

Evidence on how Chinese consumers rank these tiers is not fully consistent. Studies have documented a substantial willingness to pay for Green Food [37] and positive valuations of certified pork [4,41], while label effects interact with brand information [42] and with combinations of sustainability labels [25]. Recent market evidence suggests a growing preference for Green and Organic over Hazard-free certification [43], yet because familiarity and trust can outweigh formal stringency [20], the longer-established Green Food label may be valued as highly as, or more highly than, Organic certification. One reason for these mixed findings may be that distinguishing the tiers requires information about what each one certifies, which is difficult to infer from the logo alone. Consumers with limited acquisition ability may perceive all three as broadly similar safety assurances, whereas those with greater ability may be more likely to recognize the additional assurances of Green and Organic certification and to regard Hazard-free certification as a baseline expectation. Whether preferences across certification tiers differ systematically with consumers’ ability to acquire label information remains largely unexamined, and this is the gap the present study addresses.

## 3. Theoretical Framework and Hypotheses

Building upon the cognitive shift identified in the literature—from external information provision to internal acquisition capacity—this section develops a predictive model of consumer choice. We integrate information economics with psychometrics to formally characterize how consumers’ latent IAA shapes their preferences [37].

Random utility theory posits that consumers make choices from a set of alternatives based on the utility associated with each option [44]. Specifically, the total utility that consumer *i* derives from choosing alternative *j* can be expressed as

(1) $$U_{\mathrm{ij}}=\delta_{i}^{\prime }X_{\mathrm{ij}}+\varepsilon_{\mathrm{ij}}$$

where *X_ij_* = (*X_ij_*_1_, …, *X_ijK_*) denotes a vector of *K* product attributes (e.g., organic certification, green certification, Hazard-free certification), and *δ_i_* = (*δ_i_*_1_, …, *δ_iK_*) represents consumer *i*’s marginal utilities for these attributes, capturing preference heterogeneity. The term *ε_ij_* is a stochastic error component. Equivalently, Equation (1) can be written as

(2) $$U_{\mathrm{ij}}=\sum_{k}\delta_{\mathrm{ik}}X_{\mathrm{ijk}}+\varepsilon_{\mathrm{ij}}$$

Conventional models typically treat *δ_i_* as exogenously given or capture heterogeneity solely through random coefficients. However, in food markets characterized by complex labeling systems, consumers may encounter dispersed information channels that differ in quality and ease of access. Individuals with lower IAA may rely on limited or less informative channels, whereas those with higher IAA are better able to acquire relevant information, distinguish credible sources, and recognize meaningful differences across label attributes. In this sense, IAA constitutes a systematic source of preference heterogeneity because it affects whether attribute-related information becomes salient in utility evaluation. This implies that *δ_i_* is not fully exogenous but is partially determined by individual IAA. Accordingly, we parameterize *δ_ik_* as

*δ_ik_* = *δ*_0*k*_ + *γ_k_* ⋅ *IAA_i_* + *η_ik_* (3)

where *δ*_0*k*_ denotes the average preference for attribute *k*, *γ_k_* captures the effect of IAA on the marginal utility of attribute *k*, and *η_ik_* is an individual-specific random disturbance term. Substituting into the utility function yields

(4) $$U_{\mathrm{ij}}=\sum_{k}(\delta_{0k}+\gamma_{k}\cdot IAA_{i}+\eta_{\mathrm{ik}})X_{\mathrm{ijk}}+\varepsilon_{\mathrm{ij}}$$

This formulation indicates that IAA affects consumer utility evaluation and choice outcomes by reshaping the marginal utility associated with each attribute. Since label attributes differ in informational richness and interpretive complexity, the effect of IAA is unlikely to be uniform across labels. Information-intensive certifications such as Green and Organic convey richer production-standard and quality signals, so their incremental value is more readily identified and incorporated into utility evaluation by consumers with higher IAA. In contrast, Hazard-free certification functions primarily as a baseline safety threshold, ensuring compliance rather than signaling additional quality premiums. As IAA increases, its incremental informational gain may diminish, making it more likely to be treated as a minimum requirement rather than a differentiating quality signal.

Taken together, IAA exerts differentiated effects across attributes by shaping information acquisition and value translation. Attributes with higher informational demands exhibit increasing marginal utility, whereas low-information attributes decline in relative importance. In this way, IAA reconfigures the role of label attributes in consumer decision-making.

Based on the transition from signal provision to information acquisition, we propose the following hypotheses:

**H1** **(hierarchical channel hypothesis).** *Information channels differ systematically in their difficulty and discrimination parameters as indicators of consumers’ information acquisition ability*.

**H2** **(premiumization hypothesis).** *Higher information acquisition ability is associated with greater marginal utility from Green Food and Organic certifications*.

**H3** **(crowding-out hypothesis).** *Higher information acquisition ability is associated with lower marginal utility from the basic Hazard-free certification, which consumers with greater acquisition ability are expected to treat as a minimum requirement rather than a differentiating quality signal*.

## 4. Materials and Methods

To empirically test the relationship between IAA and consumer preferences, this study combines a structured consumer survey with a DCE to capture both the latent cognitive traits of the respondents and their hypothetical market behavior. Building on this design, we adopt a sequential empirical strategy that integrates IRT with an RPL model. Specifically, IRT is first used to recover individual-level IAA from heterogeneous information channels, after which the estimated IAA is incorporated into the RPL model to assess its impact on attribute-specific marginal utilities. This section details the experimental design, the sampling procedures, and model specifications.

### 4.1. Experimental Design and Attribute Selection

To analyze consumers’ IAA and its impact on preferences, we employed a DCE focused on the Chinese pork market. Pork provides a particularly suitable empirical setting, given its centrality to the Chinese diet, the environmental implications of pork production, and the ongoing transition from traditional wet markets to modern retail channels [45,46]. As experience-based, face-to-face evaluation is increasingly supplanted by standardized certification and labeling systems, IAA becomes more salient in shaping consumer decisions. Within this setting, the DCE offers a natural framework for capturing real-world trade-offs and identifying the marginal utility of label signals conveying different degrees of safety, quality, and sustainability assurance. To ensure a controlled decision environment and eliminate price variation across different meat cuts, we standardized the product as pork hindquarters.

Following a review of the prevailing certification landscape and consumer concerns regarding food safety and sustainability, we selected four primary attributes (Table 1). While certified labels constitute the dominant and most policy-relevant signals in the Chinese context, we extend the attribute space to include additional information cues—namely origin and drug-free claims—to better capture how different types of label information shape consumer choices in a hypothetical but policy-relevant setting.

- Food certification: This attribute serves as the primary categorical variable for our analysis, structured according to the national hierarchy: Hazard-free, Green, and Organic certifications, with no certification specified as the baseline category. The three tiers convey progressively broader benefits, ranging from basic safety assurance to enhanced quality and sustainability credentials.
- Origin information: Specified as the presence or absence of origin information. Following Yin et al. [42], we used neutral plain text to convey origin rather than naming specific regions, thereby isolating the value of traceability from regional brand bias.
- Drug-free claim: Although not yet a mainstream labeling scheme in China, we include a binary drug-free label (present/absent) to capture consumer concerns over veterinary drug residues; in the experiment, the label stated “No additives or veterinary drug residues were detected” and, unlike the three certifications, it does not imply a certified production standard, which has been salient in past food-safety incidents (e.g., clenbuterol). Introducing this attribute enables us to examine whether targeted safety claims provide additional utility beyond broad certification schemes [41,47].
- Price: We defined three levels—15, 25, and 40 yuan per 0.5 kg—based on market surveys from the study period. These levels provide the necessary variation to identify the marginal utility of price and to anchor trade-offs among attributes.

**Figure 1:**
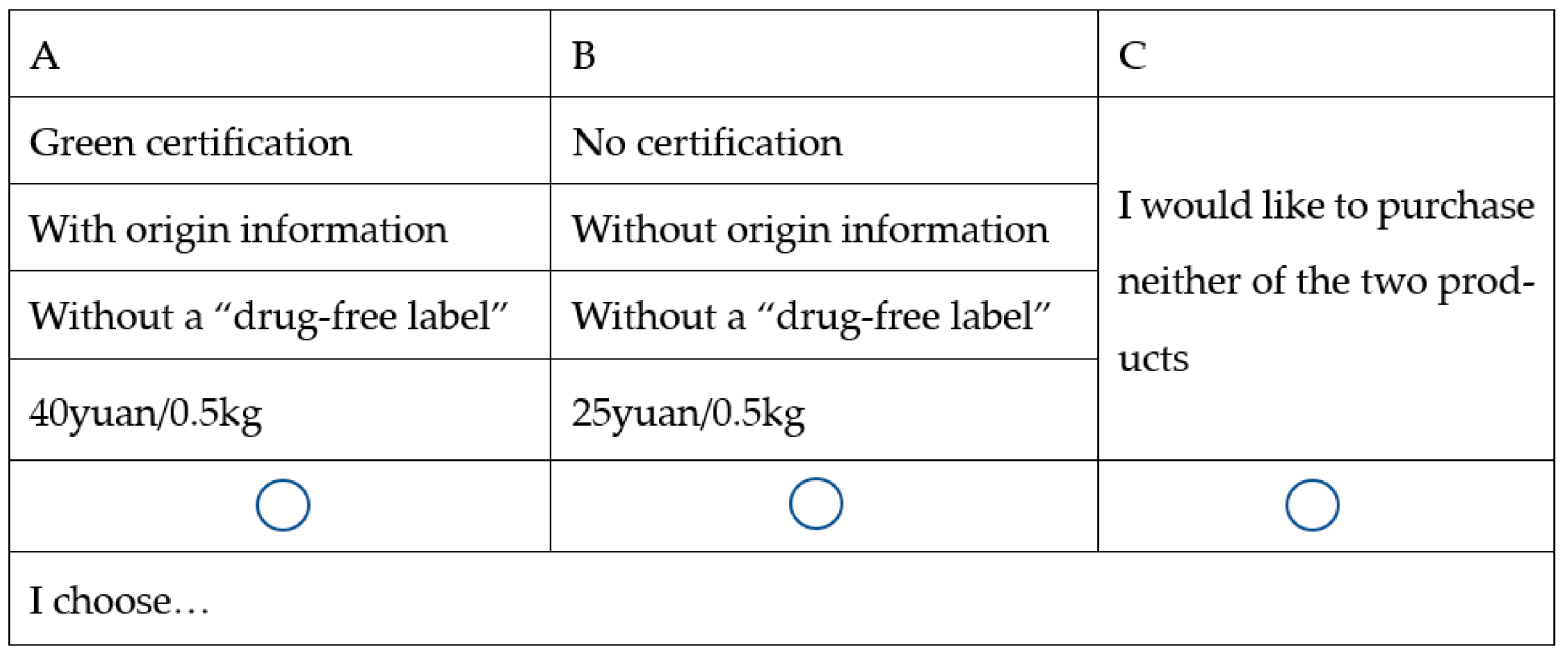
Sample choice set configuration. Note: Each set presents two multi-attribute product profiles alongside an opt-out alternative. The complete set of 12 choice sets is provided in Supplementary Materials.

The experimental design was optimized using a D-optimal partial factorial approach [48] via JMP 11.0. This procedure yielded a single questionnaire version comprising 12 choice sets (Figure 1); the complete set of choice-set configurations is provided in Supplementary Materials File S1. The design achieved a D-efficiency of 96.293, signifying a high degree of statistical power in capturing attribute trade-offs. To ensure ecological validity and minimize forced-choice bias, an opt-out alternative (Alternative C) was included in every choice set; this allows for a more realistic approximation of market behavior and provides more conservative, credible welfare estimates [33].

### 4.2. Measurement of Latent IAA and Consumer Knowledge

Additional data were collected via a structured questionnaire to facilitate the estimation of the latent IAA and to assess subjective knowledge levels. To capture the multi-dimensional nature of China’s food-label information landscape, we first identified eight primary information acquisition channels. These include digital media (online social media), broadcast media (television and radio), print media (newspapers and magazines), interpersonal networks (family, friends, and neighbors), point-of-purchase cues (packaging instructions and sales personnel), and institutional sources (community noticeboards and government departments). This taxonomy, validated through preliminary in-depth interviews, reflects the practical diffusion of label information across both traditional and digital pathways. For each channel, respondents were asked whether they could effectively obtain information about food labels (e.g., certification types and their standards) in their daily lives. Based on these responses, eight binary indicators are constructed to capture individuals’ ability to acquire and utilize label-related signals across information sources. These items serve as observed measures of IAA and are modeled in Section 4.4.1 using a two-parameter logistic IRT framework.

In addition to acquisition channels, we conducted a certification-literacy assessment to elicit respondents’ self-reported familiarity with the three national certification tiers (Hazard-free, Green, and Organic). Given the prominence of these labels in China’s domestic certification system, we measured respondents’ familiarity with the standards and procedures associated with each tier. Respondents rated their familiarity on a five-point Likert scale ranging from 1 (very unfamiliar) to 5 (very familiar). This subjective measure serves as a benchmark for the behavior-based IAA construct, enabling a contrast that sheds light on its underlying cognitive dimension.

### 4.3. Survey Implementation and Data Description

The survey was conducted between July and September 2017 across twelve representative cities in Jiangsu and Anhui provinces. These regions in East China serve as ideal locations for this study due to their well-developed markets for safety-certified foods and diverse economic profiles. To ensure regional representativeness, the sample included three distinct economic zones within each province. In Jiangsu, we surveyed the southern (Suzhou, Wuxi, Changzhou), central (Nantong, Taizhou, Yangzhou), and northern regions (Suqian, Xuzhou, Huai’an). In Anhui, the survey covered the southern (Xuancheng), central (Hefei), and northern (Bengbu) regions.

We employed proportionate sampling based on city populations. To maximize randomness during on-site recruitment, field staff utilized a systematic-intercept rule, approaching every third passerby for a face-to-face interview [4]. Eligibility was restricted to individuals aged 18 or older who had purchased pork within the previous month. From this process, 844 valid questionnaires were obtained. Informed consent was obtained from all individual participants included in the study.

As summarized in Table 2, the sample reflects the primary grocery-shopping demographic in urban China. Females constitute a slightly larger share (56.04%), and while the age range is broad, the sample skews toward younger and middle-aged adults (74.52% are under 50 years old). Given this age distribution, we examined whether IAA varied systematically with age. The correlation was weak and non-significant (Spearman’s *p* = −0.043, *p* = 0.208), suggesting that differences in acquisition ability in this sample are not driven primarily by age. The respondents are generally well-educated, with 33.89% holding at least a college degree. Economically, 65.76% of households reported an annual income above 80,000 yuan, while 12.56% reported less than 50,000 yuan. Approximately 53.79% identified as the primary household food purchaser, and 48.93% had minor children in the household.

### 4.4. Econometric Model

The empirical analysis is conducted in two distinct stages. First, we utilize IRT to derive a latent measure of consumer IAA. Second, this latent trait is integrated into an RPL model to examine how IAA shapes preference heterogeneity across food-label attributes in the pork market. The first-stage IRT model was estimated in R 4.4.1, and the second-stage RPL model in NLOGIT 5.0.

#### 4.4.1. Stage 1: The Two-Parameter Logistic IRT Model

Following Ghadim et al. [49], we estimated a two-parameter IRT model with a logistic link to assess consumers’ IAA based on their use of various information acquisition channels. Specifically, let *y_ik_* be a binary indicator equal to 1 if consumer *i* utilizes information channel *k*, and 0 otherwise. Then *P*(*y_ik_* = 1|*θ_i_*) denotes the probability that consumer *i* utilizes information channel *k*, conditional on their latent IAA, *θ_i_*. The model is expressed as

(5) $$P(y_{\mathrm{ik}}=1|\theta_{i})=\frac{\exp[\alpha_{k}(\theta_{i}-\beta_{k})]}{1+\exp[\alpha_{k}(\theta_{i}-\beta_{k})]}$$

where *θ_i_* is assumed to follow a standard normal distribution. The parameter *α_k_* is the discrimination parameter, capturing how effectively channel *k* distinguishes between individuals with different levels of ability, while *β_k_* is the difficulty parameter, representing the level of ability required to effectively access or utilize information from channel *k*. The model parameters are estimated using marginal maximum likelihood (MMLE). Individual-level latent ability *θ_i_* is then recovered using expected a posteriori (EAP) estimation and used as the measure of IAA in the subsequent analysis.

#### 4.4.2. Stage 2: RPL Model

In the second stage of our econometric strategy, we integrate the latent IAA scores (*θ_i_*) derived from Stage 1 into the choice model. This allows us to observe not just general preferences, but how IAA modulates a consumer’s preference for specific food-label attributes.

Given that each respondent makes repeated choices across multiple choice situations in the discrete choice experiment, we introduce a situation index *t* to capture within-individual variation across choice tasks. The utility that consumer *i* derives from alternative *j* in the choice situation *t* is specified as

(6) $$U_{\mathrm{ijt}}=\sum_{k}\delta_{\mathrm{ik}}X_{\mathrm{ijkt}}+\varepsilon_{\mathrm{ijt}}=\sum_{k}(\delta_{0k}+\gamma_{k}\cdot \theta_{i}+\eta_{\mathrm{ik}})X_{\mathrm{ijkt}}+\varepsilon_{\mathrm{ijt}}$$

Conditional on the individual-specific parameter vector *δ_i_*, the probability that consumer *i* chooses alternative *j* in choice situation *t* follows the standard logit form:

(7) $$P_{\mathrm{ijt}}(\delta_{i})=\frac{\exp(\delta_{i}^{\prime }X_{\mathrm{ijt}})}{\sum_{m}\exp(\delta_{i}^{\prime }X_{\mathrm{imt}})}$$

where *m* indexes all alternatives in the choice set of situation *t*.

Since the preference parameters *δ_i_* include random components *η_ik_*, we assume that they follow a normal distribution across individuals. The unconditional choice probability is therefore obtained by integrating over the distribution of *δ_i_*:

(8) $$P_{\mathrm{ijt}}=\int \frac{\exp(\delta_{i}^{\prime }X_{\mathrm{ijt}})}{\sum_{m}\exp(\delta_{i}^{\prime }X_{\mathrm{imt}})}f(\delta_{i})d\delta_{i}$$

This specification characterizes the RPL model and accommodates preference heterogeneity across individuals. We use this framework to estimate consumer preferences for food-label attributes in the pork market and to examine how these valuations are modulated by latent IAA.

## 5. Results

### 5.1. Estimation Results of the IRT Model

#### 5.1.1. Item Parameter Estimation: Identifying the “Information Bottleneck”

The results of the two-parameter logistic IRT model, presented in Table 3, provide the foundational empirical basis for H1 by identifying the difficulty parameters (*β*) and the discrimination parameters (*α*) across the eight acquisition channels.

As detailed in Table 3, the discrimination and difficulty parameters for all eight channels are statistically significant at the 5% level or better, confirming their empirical relevance in defining the latent structure of consumer IAA. The results point to an information bottleneck characterized by a divergence between ease of access and discrimination. Digital channels (online social media) exhibit the lowest difficulty threshold (*β* = 0.190), indicating that even respondents with below-average IAA are likely to obtain label information from it. At the same time, online social media has a relatively high discrimination parameter (*α* = 1.038), second only to newspapers and magazines, meaning that the likelihood of obtaining label information from this channel rises steeply as IAA increases. This combination suggests a digital paradox: although digital channels are widely accessible, they differentiate sharply between consumers with different levels of acquisition ability. In this sense, differences in access to label information through digital channels may stem less from physical access than from consumers’ ability to make effective use of these channels.

In contrast, traditional print media (newspapers and magazines) exhibit the highest discrimination (*α* = 1.255), making it the channel that differentiates most sharply between consumers of different ability. Institutional channels, such as government departments and community noticeboards, show extreme difficulty (*β* ≥ 3.000), indicating that while they provide standardized safety signals, they remain largely underutilized by the general public and are used mainly by respondents with the highest IAA.

This structural divergence across channel types—where digital platforms are easy to access but highly discriminating and institutional sources remain high-barrier—supports H1 (The hierarchical channel hypothesis). It indicates that information channels are not homogeneous and that market agency is filtered through distinct technical and cognitive entry barriers.

#### 5.1.2. Distribution of IAA and the “Knowledge Graduation” Effect

The distribution of the estimated IAA scores indicates that acquisition ability is limited for a majority of respondents: 29.86% (*n* = 252) have scores between −1 and −0.5, 22.16% (*n* = 187) between −0.5 and 0, 26.30% (*n* = 222) between 0 and 0.5, and 13.98% (*n* = 118) between 0.5 and 1. In total, 52.02% of respondents fall at or below zero, while only a small segment (7.70%, *n* = 65) has high acquisition ability (IAA > 1).

**Figure 2:**
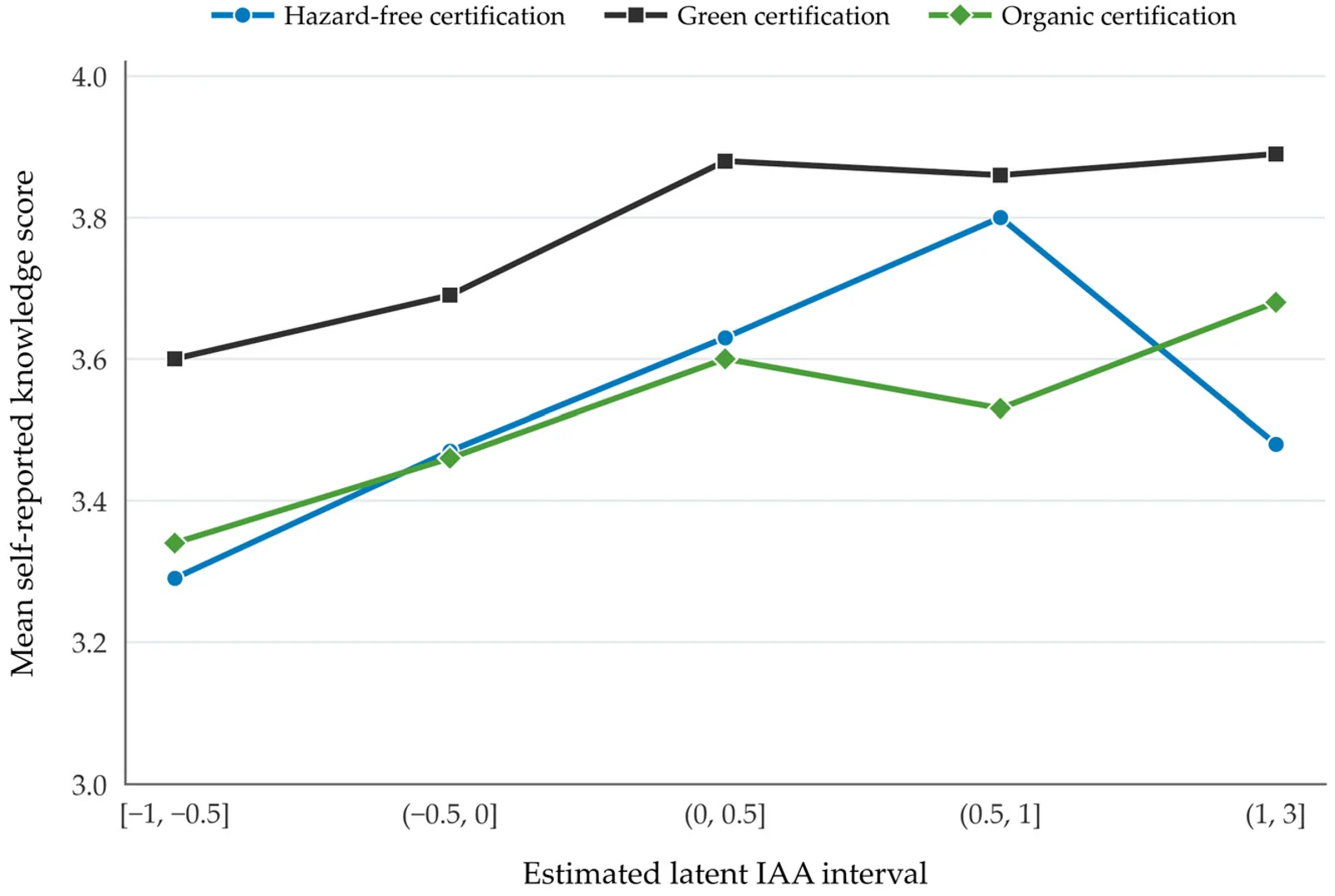
Comparison of latent IAA and subjective knowledge of food certifications. (Note: The vertical axis represents the mean self-reported knowledge score on a Likert scale for Hazard-free, Green, and Organic certifications across estimated IAA intervals).

To validate these latent scores, Figure 2 illustrates the relationship between IAA and self-reported knowledge across the three certification tiers. While a consistent positive gradient exists for Green and Organic labels, the data suggest a knowledge graduation effect: within the highest IAA cohort, self-reported knowledge of the baseline Hazard-free certification is lower than in the adjacent cohort. This suggests that as consumers attain higher levels of IAA, they may engage in cognitive resource re-allocation. Rather than accumulating exhaustive knowledge of all market signals, high-ability consumers may shift their attention away from entry-level safety signals (Hazard-free) toward the more complex, credence-heavy requirements of high-tier certifications. This pattern may reflect a gradual transition in which consumers with higher IAA become less exclusively oriented toward risk avoidance and more strongly engaged with premium food attributes, including the quality and environmental credentials embedded in higher-tier labels.

### 5.2. Consumer Preferences for Food-Label Attributes

Table 4 reports the RPL estimation results, which reveal several plausible patterns regarding the Chinese pork market. As expected, the price coefficient is negative, aligning with basic economic intuition. The opt-out coefficient is negative and significant (−1.924), indicating that respondents generally preferred to purchase one of the labeled products rather than neither. The coefficients for Hazard-free, Green, and Organic certifications are all positive and significant at the 1% level. Because the certification attribute is effects-coded, the implied coefficient for the no-certification level is −1.322 (the negative sum of the three certification coefficients), so each certification increases utility substantially relative to an uncertified product. The coefficients for Green (0.564) and Organic (0.557) are nearly identical and almost three times as large as that for Hazard-free certification (0.201). The resulting ranking—Hazard-free < Organic ≤ Green—departs from the institutional hierarchy, in which Organic is the most stringent tier. This reflects the broader trend of consumption upgrading [37] alongside the specific maturity of the Chinese market. While the domestic organic sector remains relatively small and poorly understood, the Green certification has benefited from a longer market presence and established institutional trust since its inception in 1990 [42]. Although the difference between the Green and Organic coefficients is small, the slightly higher valuation of Green suggests that while consumers prioritize enhanced safety and quality, they may perceive Green food as the optimal utility-maximizing signal—representing a rational trade-off between rigorous safety standards and price premiums. In contrast, Organic certification may be met with skepticism due to perceived over-pricing or lack of familiarity, a phenomenon typical of transitioning agricultural markets [42]. These findings align with the proficiency levels and knowledge gradients observed in Figure 2.

Regarding origin labels, consumers exhibit a strong preference for pork carrying geographical information (*δ* = 0.599, *p* < 0.01), consistent with prior findings [50]. Although the study design intentionally withheld specific origins to avoid location-driven biases [42], origin labels still enable consumers to infer traceability and regional familiarity, thereby reducing information asymmetry [51]. Furthermore, the drug-free claim is positive and significant at the 1% level (*δ* = 0.390, *p* < 0.01). Given the historical context of improper veterinary pharmaceutical use and insufficient compliance with administration protocols in China, residues pose a notable public health risk [52]. A drug-free claim signals safety and compliance, which may make such products more attractive to risk-averse consumers. This finding suggests that drug-free labeling, although not yet widely adopted, may provide a promising basis for product differentiation and market development within a broader transition toward more transparent and credence-based food markets.

### 5.3. The Impact of IAA on Consumer Preferences: Premiumization and Crowding-Out

The interaction terms in Table 4 show how preferences vary with IAA. The interactions between IAA and Green certification (*γ* = 0.103, *p* < 0.05) and Organic certification (*γ* = 0.096, *p* < 0.05) are positive and significant at the 5% level, indicating that consumers with higher IAA attach greater utility to these higher-standard certifications; this supports H2. The interaction with Hazard-free certification is negative (*γ* = −0.073) but significant only at the 10% level and not at the 5% level (*p* < 0.10), so the evidence for H3 is weaker. Taken together, the opposite signs of the interactions suggest that consumers with greater information acquisition ability place relatively greater value on Green and Organic certifications and relatively less value on the entry-level safety signal.

One interpretation is that higher IAA enables consumers to perceive Green and Organic labels as signals of superior environmental and health attributes that go beyond the baseline safety assurances of Hazard-free certification. This pattern is consistent with a partial crowding-out of the baseline label, in which consumers with higher IAA appear to treat Hazard-free certification as a minimum requirement rather than as a differentiating signal. IAA may therefore act not only as a facilitator of access but also as a condition for the effective functioning of a multi-tiered label market.

Regarding non-certification attributes, the interaction between origin labels and IAA is not statistically significant. This aligns with prior research suggesting that origin preferences are largely anchored in purchasing habits and regional familiarity [50]. One explanation is that an origin label is a low-entry-barrier signal; it represents a simple geographic heuristic that does not require high IAA. Conversely, the drug-free label interaction with IAA is significantly positive (*γ* = 0.073, *p* < 0.01). Since drug-free claims are often technologically embedded within broader certification frameworks rather than presented as standalone signals [41], their perceived value may depend on the consumer’s ability to decode the underlying labeling system. Higher IAA may therefore strengthen consumer confidence in these technical attributes, which is consistent with the higher marginal utility of drug-free claims among consumers with greater acquisition ability.

### 5.4. Results of Heterogeneity Analysis

Identifying sensitive consumer segments is essential for optimizing the efficiency of resource allocation in support of agricultural sustainability transitions [21]. To this end, we conducted a heterogeneity analysis across two critical dimensions: prior experience with quality-safety incidents and the presence of minors in the household (Table 5).

For consumers without prior experience of pork-safety incidents, the interaction coefficients largely mirror the full-sample results in Table 4: IAA is positively associated with the valuation of Green (*γ* = 0.124, *p* < 0.01), Organic (*γ* = 0.082, *p* < 0.05) and Drug-free (*γ* = 0.078, *p* < 0.01) attributes and negatively associated with Hazard-free certification (*γ* = −0.085, *p* < 0.10). For consumers who reported experiencing a pork-safety incident, only the interaction between Organic certification and IAA is significant (*γ* = 0.241), at the 10% level rather than the 5% level (*p* < 0.10). This contrast suggests that prior experience of a safety incident may alter how consumers evaluate label signals, with more capable consumers in this group placing relatively more weight on the most stringent certification. Nevertheless, the absence of significant interactions for the other attributes should not be read as indicating that these attributes are not valued: their main effects remain positive and significant in this group, and the smaller size of the subgroup may limit the precision of the interaction estimates.

Regarding household composition, households without minors exhibit no significant interaction terms, which may indicate that IAA plays a smaller role in label valuation when the perceived consequences of consumption are lower. In contrast, for households with children, the interactions of IAA with Green (*γ* = 0.158, *p* < 0.05), Drug-free (*γ* = 0.139, *p* < 0.05), Organic (*γ* = 0.096, *p* < 0.10) and origin information (*γ* = 0.065, *p* < 0.10) are positive. These consumers appear to use their IAA more effectively to navigate the labeling regime, reflecting the heightened risk sensitivity and intergenerational responsibility associated with child-rearing [53,54]. For this group, higher IAA is associated with stronger preferences for higher-standard label attributes, suggesting that informational competence may help parents mitigate perceived risks to vulnerable household members.

## 6. Discussion

### 6.1. Comparison with Previous Studies

Our findings are consistent with, and extend, several strands of previous research. The channel results echo studies of the second-level digital divide, which show that once access becomes widespread, inequalities shift to differences in skills and use [30,31]; in the same way, greater digital transparency does not by itself equalize consumers’ access to label information. Like Abdul-Salam and Phimister [34], who used IRT to measure farmers’ access to information, we find that information sources are far from equivalent indicators. The high difficulty of government channels also contrasts with evidence that Chinese consumers value government-backed certification most highly [21,22], suggesting that the signals consumers trust most are not necessarily those they can access most easily.

The label results are in line with earlier choice experiments showing that certification increases the utility of pork in urban China [4,41] and with the substantial willingness to pay for Green Food reported by Yu et al. [37]. That Green certification is valued at least as highly as the stricter Organic certification supports the view that familiarity and trust in a label can matter more than its formal stringency [20]. The positive association between IAA and the valuation of Green and Organic certifications extends work showing that knowledge and understanding shape attitudes toward, and the use of, organic and sustainability labels [19,29]: our results suggest that the ability to acquire label information, not only the stock of knowledge, matters. They also refine the observation of Sameshima and Akamatsu [9] that even consumers concerned about food hygiene may lack sufficient knowledge of food safety: the gap between concern and effective label use appears to reflect not only motivation but also differences in consumers’ ability to acquire credible information. The weaker negative association with Hazard-free certification fits evidence that more knowledgeable consumers concentrate on more diagnostic information [28]. Finally, the stronger associations among households with minors are consistent with the finding that having children at home increases the likelihood of buying organic food [29].

### 6.2. Policy Implications

The empirical evidence suggests that information policy must account for the inherent heterogeneity in consumer acquisition ability. Given that online social media has a high discrimination parameter despite low access barriers, expanding digital transparency alone is insufficient to support healthier and more sustainable food choices. Policymakers should prioritize high-efficiency channels while simultaneously lowering the cognitive thresholds for institutional information. This requires simplifying the presentation of high-discrimination safety data—such as government department announcements—into standardized, easily interpretable formats that reduce the latent ability required for a consumer to derive utility from the signal.

Furthermore, the pattern consistent with label crowding-out suggests a need for a segmented approach to labeling policy. China’s multi-tiered framework offers a pragmatic, staged mechanism for other developing economies where entry- and mid-tier signals, such as Hazard-free and Green labels, can help safeguard agricultural safety in emerging markets. However, resources should be allocated based on the maturity of the target segment. For the majority of the population with relatively low IAA, maintaining the credibility of these mid-tier labels remains the primary public health priority. Conversely, for the high-IAA segment, policy should focus on bridging the knowledge gap regarding organic standards [42], as these consumers appear to value baseline signals less. This pattern is also consistent with the ongoing replacement of Hazard-free certification by a compulsory certificate of compliance [55]: for more capable consumers, baseline safety is increasingly a minimum requirement rather than a basis for differentiation. At the same time, our findings indicate that more technical safety attributes—such as drug-free claims—represent underexploited market potential. Relative to conventional labels, these attributes are typically embedded within existing certification systems, yet their policy value lies in offering more granular signaling instruments for product differentiation. Accordingly, regulators and standard-setting bodies could make such attributes more explicit and standardized in their presentation. Doing so would enable high-IAA consumers to more readily identify core safety and quality information without adding institutional complexity, while expanding the market scope for higher-standard products.

Finally, targeted behavioral interventions and communication strategies must be prioritized according to household risk profiles. For consumers affected by prior safety incidents, our more tentative evidence suggests that communication emphasizing high-stringency certifications may be more effective [25,41]. This conclusion should, however, be confirmed with larger samples. In contrast, households with minors represent the most responsive segment for proactive outreach; these families appear to use their IAA most actively to protect vulnerable members, suggesting that tailored safety communication and behavioral nudges for this group may yield the highest return on choice-architecture governance and in the market acceptance of more sustainability-oriented food options [53].

### 6.3. Limitations and Future Research

Despite its contributions, this study is subject to several limitations that provide avenues for future inquiry. First, while the choice experiment focused on three primary label categories to maintain experimental validity and reduce respondent fatigue, the landscape of food production and agricultural sustainability is rapidly evolving. Future research should expand this framework to include emerging eco-labels, such as carbon footprint signaling and biodiversity-friendly certifications, to assess how IAA moderates the adoption of second-generation green attributes.

Second, although the DCE is a robust method for eliciting preferences, a potential hypothetical bias remains between stated preferences and actual purchasing behavior. Future work would benefit from integrating e-commerce purchase data, real-world scanner data, or longitudinal tracking to address these experimental biases and explore the long-term evolution of IAA across digital retail platforms.

Third, the survey was conducted among urban consumers in Jiangsu and Anhui, two provinces with relatively well-developed markets for certified food, and younger and better-educated consumers are somewhat over-represented in the sample. The findings are therefore most informative for urban consumers in more developed regions. Future studies could extend the analysis to rural and less developed areas, where label environments and consumers’ acquisition ability may differ.

Finally, we acknowledge that the primary data for this study were collected in 2017. While the digital landscape has since advanced, this period represents a unique and critical baseline in China’s food safety trajectory. The year 2017 serves as a vital inflection point where the mass adoption of mobile digital channels intersected with the formalization of the national multi-tiered labeling regime. Thus, these results offer a foundational benchmark for understanding the structural roots of the patterns observed for digital channels before the market reached its current state of information saturation. Future comparative studies using more recent data could build on this 2017 baseline to examine how consumers’ IAA evolves with technological maturity, and how such changes reshape preferences across baseline, mid-tier, and premium safety labels.

## 7. Conclusions

This study examined how consumers’ information acquisition ability relates to their preferences for pork-label attributes, combining an IRT-based measure of IAA with a discrete choice experiment among 844 consumers in Jiangsu and Anhui provinces. Three findings stand out. First, information channels play distinct roles in consumers’ acquisition of label information: online social media is the most accessible channel but differentiates sharply between consumers of different ability, whereas institutional channels such as government departments and community noticeboards are the most difficult to use and reach mainly the small group of consumers with the highest IAA. Second, all label attributes increase consumer utility, with Green and Organic certifications valued most highly; consumers with higher IAA place greater value on Green, Organic and drug-free attributes and, to a lesser extent, less value on the entry-level Hazard-free certification. Third, these associations are most evident among households with minors, while consumers who had experienced a pork-safety incident appear to focus more selectively on Organic certification. Together, these results show that the effectiveness of food labels depends not only on the information supplied but also on consumers’ capacity to acquire and interpret it. Making institutional label information easier to access and understand, and tailoring communication to families with children, are promising ways to strengthen market incentives for safer and more sustainable pork production.

## Figures and Tables

**Table 1:** Food label attributes and levels used in the discrete choice experiment.

Attribute	Level	Effect Coding
Food certification	“Hazard-free certification” (Hazard-free)	Hazard-free = 1; Green = 0; Organic = 0
	“Green certification” (Green)	Hazard-free = 0; Green = 1; Organic = 0
	“Organic certification” (Organic)	Hazard-free = 0; Green = 0; Organic = 1
	No certification (Nocert)	Hazard-free = −1; Green = −1; Organic = −1
Origin information	With origin information (Origin)	Origin =1
	Without origin information (Noorigin)	Noorigin = −1
Drug-free claim	With “drug-free label” (Drugfree)	Drugfree = 1
	Without “drug-free label” (Nodrugfree)	Nodrugfree = −1
Price	15 yuan/0.5 kg	Price = 15; Price = 25; Price = 40
	25 yuan/0.5 kg	
	40 yuan/0.5 kg	

**Table 2:** Descriptive statistics of respondent socio-demographic characteristics.

Variables	Category	Observations	Percentage (%)
Gender	Male	371	43.96
	Female	473	56.04
Age	18–29 years old	255	30.21
	30–39 years old	151	17.89
	40–49 years old	223	26.42
	50–59 years old	134	15.88
	60 years old and above	81	9.60
Education	Junior high school and below	237	28.08
	Senior high school (including professional high school)	208	24.64
	College	113	13.39
	Bachelor	240	28.44
	Postgraduate and above	46	5.45
Annual household income	50,000 yuan and below	106	12.56
	50,000–80,000 yuan	183	21.68
	80,000–100,000 yuan	230	27.25
	100,000 yuan and above	325	38.51
Minor children in the family	Yes	413	48.93
	No	431	51.07
The main buyers of household food	Yes	454	53.79
	No	390	46.21

**Table 3:** Parameter estimates for the two-parameter logistic IRT model.

Type of Information Channel	Discrimination Parameter (α)	S.E.	Difficulty Parameter (β)	S.E.
Online social media	1.038 ***	0.227	0.190 **	0.086
Television and radio	0.459 ***	0.143	0.621 **	0.237
Newspapers and magazines	1.255 ***	0.280	1.355 ***	0.204
Family, friends, and neighbors	0.303 **	0.134	2.861 **	1.237
Packaging instructions	0.681 ***	0.211	3.378 ***	0.912
Community noticeboards	0.484 **	0.190	4.574 **	1.664
Sales personnel	0.449 ***	0.139	1.425 ***	0.438
Government departments	0.881 ***	0.246	3.000 ***	0.678

Note: *** and ** indicate significance at the 1% and 5% levels, respectively. S.E.: standard error.

**Table 4:** Econometric estimates of consumer preferences for pork-label attributes.

Variables	Estimation Coefficient	S.E.	Variables	Estimation Coefficient	S.E.
Main effects					
Opt Out	−1.924 ***	0.048	Organic	0.557 ***	0.025
Price	−0.045 ***	0.002	Origin	0.599 ***	0.017
Hazard-free	0.201 ***	0.027	Drug-free	0.390 ***	0.015
Green	0.564 ***	0.028			
Interaction term					
Hazard-free × IAA	−0.073 *	0.043	Origin × IAA	0.039	0.026
Green × IAA	0.103 **	0.045	Drug-free × IAA	0.073 ***	0.024
Organic × IAA	0.096 **	0.039			
log likelihood	−8851.34		AIC	17,726.7	
Participants	844		Obs	10,128	

Note: ***, **, and * indicate significance at the 1%, 5%, and 10% levels, respectively. AIC: Akaike information criterion.

**Table 5:** Heterogeneity analysis by safety-incident experience and household minors.

Variables	Whether They Experienced a Pork Safety Incident		Whether There Are Minors in the Household	
	Experience	No-Experience	Minors	No-Minors
Opt Out	−2.088 ***	−1.907 ***	−1.87 ***	−2.00 ***
PRICE	−0.054 ***	−0.044 ***	−0.046 ***	−0.043 ***
Hazard-free	0.229 ***	0.199 ***	0.217 ***	−0.190 ***
Green	0.549 ***	0.566 ***	0.593 ***	0.548 ***
Organic	0.621 ***	0.551 ***	0.534 ***	0.576 ***
Origin	0.522 ***	0.609 ***	0.604 ***	0.596 ***
Drug-free	0.377 ***	0.392 ***	0.388 ***	0.397 ***
Hazard-free × IAA	−0.053	−0.085 *	−0.030	−0.124
Green × IAA	0.090	0.124 ***	0.158 **	0.054
Organic × IAA	0.241 *	0.082 **	0.096 *	0.079
Origin × IAA	0.064	0.035	0.065 *	0.004
Drug-free × IAA	0.028	0.078 ***	0.139 **	0.001
log likelihood	−872.49	−7971.97	−4591.86	−4256.12
AIC	1769.0	15,967.9	9207.7	8536.2
Participants	83	761	413	431
Obs	996	9132	4956	5172

Note: ***, **, and * indicate significance at the 1%, 5%, and 10% levels, respectively. AIC: Akaike information criterion.

## Data Availability

The original contributions presented in the study are included in the article/supplementary materials, further inquiries can be directed to the corresponding authors.

## Supplementary Materials

The following supporting information can be downloaded at: https://www.mdpi.com/article/10.3390/foods15213996/s1, File S1: Choice-set configurations of the discrete choice experiment.

## Abbreviations

DCE	Discrete Choice Experiment
IAA	Information Acquisition Ability
IRT	Item Response Theory
MMLE	Marginal Maximum Likelihood
EAP	Expected A Posteriori
RPL	Random Parameters Logit
