# Supplementary material for: Information Acquisition Ability and Consumer Preferences for Food-Label Attributes: Evidence from China’s Pork Market
Source: Foods. 2026 Nov 7;15(21):3996. doi: 10.3390/foods15213996 (PMC13763410; doi:10.3390/foods15213996)
Supplement: Supplementary file 1 [file foods-15-03996-s001.zip › foods-4583562-supplementary.pdf]

**Supplementary Materials File S1: Choice-set configurations of the discrete choice experiment**

**Choice set S1**

| A | B | C |
|---|---|---|
| Organic certification | No certification | I would like to purchase neither of the two products |
| With origin information | Without origin information | |
| Without a “drug-free label” | With a “drug-free label” | |
| 40yuan/0.5kg | 25yuan/0.5kg | |
| ○ | ○ | ○ |

**Choice set S2**

| A | B | C |
|---|---|---|
| Green certification | GRS certification | I would like to purchase neither of the two products |
| Without origin information | With origin information | |
| With a “drug-free label” | Without a “drug-free label” | |
| 40yuan/0.5kg | 15yuan/0.5kg | |
| ○ | ○ | ○ |

**Choice set S3**

| A | B | C |
|---|---|---|
| Organic certification | GRS certification | I would like to purchase neither of the two products |
| With origin information | Without origin information | |
| Without a “drug-free label” | Without a “drug-free label” | |
| 40yuan/0.5kg | 25yuan/0.5kg | |
| ○ | ○ | ○ |

**Choice set S4**

| A | B | C |
|---|---|---|
| GRS certification | Organic certification | I would like to purchase neither of the two products |
| With origin information | With origin information | |
| Without a “drug-free label” | With a “drug-free label” | |
| 40yuan/0.5kg | 25yuan/0.5kg | |
| ◯ | ◯ | ◯ |

**Choice set S5**

| A | B | C |
|---|---|---|
| Green certification | No certification | I would like to purchase neither of the two products |
| Without origin information | Without origin information | |
| Without a “drug-free label” | Without a “drug-free label” | |
| 15yuan/0.5kg | 40yuan/0.5kg | |
| ◯ | ◯ | ◯ |

**Choice set S6**

| A | B | C |
|---|---|---|
| GRS certification | GRS certification | I would like to purchase neither of the two products |
| Without origin information | Without origin information | |
| With a “drug-free label” | Without a “drug-free label” | |
| 40yuan/0.5kg | 15yuan/0.5kg | |
| ◯ | ◯ | ◯ |

**Choice set S7**

| A | B | C |
|---|---|---|
| Organic certification | Green certification | I would like to purchase neither of the two products |
| Without origin information | With origin information | |
| Without a “drug-free label” | With a “drug-free label” | |
| 15yuan/0.5kg | 25yuan/0.5kg | |
| ◯ | ◯ | ◯ |

**Choice set S8**

| A | B | C |
|---|---|---|
| Organic certification | GRS certification | I would like to purchase neither of the two products |
| With origin information | Without origin information | |
| Without a “drug-free label” | With a “drug-free label” | |
| 25yuan/0.5kg | 15yuan/0.5kg | |
| ◯ | ◯ | ◯ |

**Choice set S9**

| A | B | C |
|---|---|---|
| Green certification | Organic certification | I would like to purchase neither of the two products |
| Without origin information | Without origin information | |
| Without a “drug-free label” | With a “drug-free label” | |
| 25yuan/0.5kg | 15yuan/0.5kg | |
| ◯ | ◯ | ◯ |

**Choice set S10**

| A | B | C |
|---|---|---|
| No certification | Organic certification | I would like to purchase neither of the two products |
| With origin information | Without origin information | |
| With a “drug-free label” | With a “drug-free label” | |
| 15yuan/0.5kg | 25yuan/0.5kg | |
| ○ | ○ | ○ |

**Choice set S11**

| A | B | C |
|---|---|---|
| GRS certification | Green certification | I would like to purchase neither of the two products |
| With origin information | Without origin information | |
| With a “drug-free label” | With a “drug-free label” | |
| 25yuan/0.5kg | 15yuan/0.5kg | |
| ○ | ○ | ○ |

**Choice set S12**

| A | B | C |
|---|---|---|
| Green certification | GRS certification | I would like to purchase neither of the two products |
| With origin information | Without origin information | |
| Without a “drug-free label” | Without a “drug-free label” | |
| 15yuan/0.5kg | 25yuan/0.5kg | |
| ○ | ○ | ○ |
